# An electromechanical Ising Hamiltonian

**DOI:** 10.1126/sciadv.1600236

**Published:** 2016-06-24

**Authors:** Imran Mahboob, Hajime Okamoto, Hiroshi Yamaguchi

**Affiliations:** Nippon Telegraph and Telephone Corporation, Basic Research Laboratories, Atsugi-shi, Kanagawa 243-0198, Japan.

**Keywords:** Electromechanical resonator, phonon, nonlinear, parametric resonance, nondegenerate parametric amplification, simulator, Ising Hamiltonian

## Abstract

Solving intractable mathematical problems in simulators composed of atoms, ions, photons, or electrons has recently emerged as a subject of intense interest. We extend this concept to phonons that are localized in spectrally pure resonances in an electromechanical system that enables their interactions to be exquisitely fashioned via electrical means. We harness this platform to emulate the Ising Hamiltonian whose spin 1/2 particles are replicated by the phase bistable vibrations from the parametric resonances of multiple modes. The coupling between the mechanical spins is created by generating two-mode squeezed states, which impart correlations between modes that can imitate a random, ferromagnetic state or an antiferromagnetic state on demand. These results suggest that an electromechanical simulator could be built for the Ising Hamiltonian in a nontrivial configuration, namely, for a large number of spins with multiple degrees of coupling.

## Teaser

The phonons localized in a mechanical resonator can be electrically manipulated to emulate the Ising Hamiltonian.

## INTRODUCTION

Physical simulators have emerged as a novel means for solving problems in quantum physics that are beyond the capacity of classical computers (*1*, *2*). This notion can also be applied to mathematical problems, for example, the Ising Hamiltonian used in describing a spin glass, which is characterized as a nondeterministic polynomial-time (NP)–hard problem (*3*). However, despite this formidable challenge, the ground-state spin configuration of an Ising system could yield solutions to optimization problems that can be mapped onto its spin lattice, and thus, its efficient extraction is highly desired (*4*). One approach to rapidly determine the ground state of the Ising Hamiltonian is to use quantum annealing where quantum tunneling is harnessed to search the energy landscape corresponding to a given problem programmed into the underlying spin lattice (*5*–*7*). Recently, an alternative and apparently classical approach to this problem has emerged where a time-multiplexed optical parametric resonator network is programmed with the Ising Hamiltonian. The ground state is then determined by slowly activating the network, which preferentially resonates in its global potential minima (*8*, *9*). Here, a variation of this concept is developed with phonons in a frequency-multiplexed electromechanical parametric resonator (*10*, *11*), and we show that this platform could readily be extended to multiple parametric resonances with arbitrary degrees of coupling, namely, all the ingredients necessary to solve the Ising Hamiltonian in a nontrivial configuration (*12*–*14*).

The Ising model conceived using the tools of statistical physics describes ferromagnetism, and in the absence of a magnetic field, its Hamiltonian is given by $-\sum_{ik}^{N}J_{ik}\sigma_{i}\sigma_{k}$ with *N* particles each having two spin states σ_*i*_ = ± 1 where the coupling between the *i*th and *k*th particles is parameterized by *J*_*ik*_. In the first steps to building a phonon-based simulator, the fundamentals of the Ising Hamiltonian need to be replicated in the electromechanical domain, namely, a mechanical spin that encodes σ, multiple mechanical spins that play the role of an *N* particle bath, and finally coupling between the mechanical spins *J* whose magnitude and polarity can be controlled and extended beyond nearest-neighbor particles.

## RESULTS

### Mapping the electromechanical system onto the Ising Hamiltonian

**Fig. 1:**
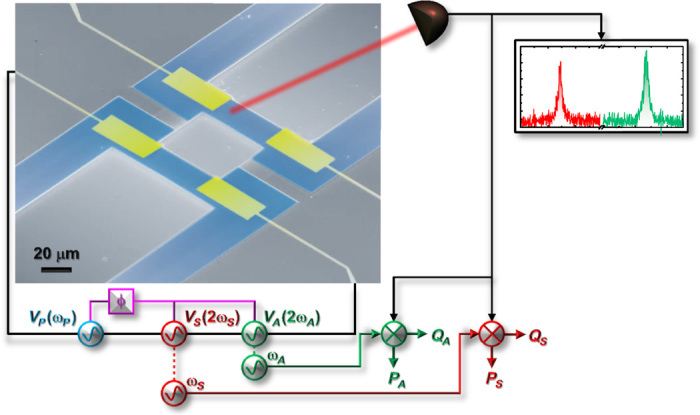
The electromechanical system. A false-color electron micrograph of the coupled mechanical resonators sustaining phonons in symmetric and asymmetric vibration modes. The measurements were performed at room temperature (300 K) and in a high vacuum (10^−6^ mbar), and the mechanical vibrations were detected using a laser interferometer, which was demodulated either in a spectrum analyzer or in a phase-sensitive detector using the heterodyne mixing setup detailed in the circuit schematic. Five signal generators were used, with two piezoelectrically activating the parametric resonances at 2ω_*n*_, two generating the reference signals for the phase-sensitive detectors at ω_*n*_, and one creating the coupling between the parametric resonances via parametric down-conversion when activated at $\omega_{𝒫}$.

**Fig. 2:**
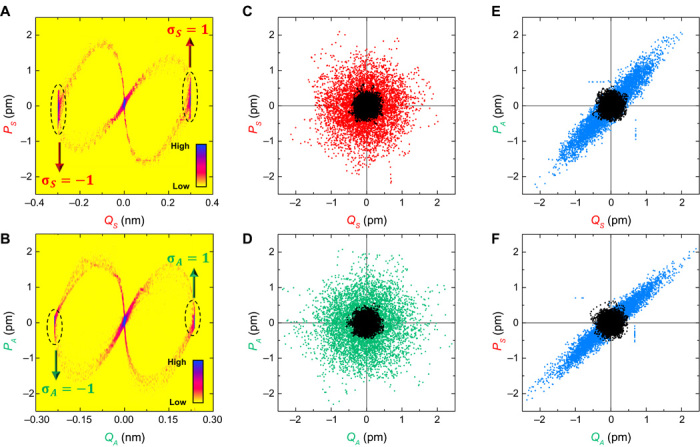
Spins and spin coupling. (**A** and **B**) The occupation probabilities of the parametric resonances are measured by periodically switching on and off their excitation with *V*_*S*_(2ω_*S*_) = 2.0 V_PP_ and *V*_*A*_(2ω_*A*_) = 3.0 V_PP_, respectively, and measuring the resultant evolution of their in-phase and quadrature components of motion. The phase portraits reconstructed from this measurement, from 2000 samples, indicate that when a parametric resonance is activated, each thermally occupied mode at the origin evolves to one of the two available oscillating states (dashed ovals) with a π phase separation via a single trajectory. When the parametric resonance is deactivated, the oscillating mode returns to the origin via another trajectory, which does not overlap with the upward trajectory (*21*). (**C** and **D**) Phase portraits corresponding to the thermal fluctuations of the symmetric and asymmetric mode as a function of $V_{𝒫}(\omega_{𝒫})=0V_{\text{PP}}$ (black points) and 1.3 V (red and green points, respectively). (**E** and **F**) The phase portraits reconstructed from the in-phase component of one of the modes versus the quadrature component of the other mode and vice versa, from the data in (C) and (D), again as a function of $V_{𝒫}(\omega_{𝒫})=0V_{\text{PP}}$ (black points) and 1.3 V_PP_ (blue points), reveal squashed distributions, implying that the motion of both modes has become correlated.

The prototype electromechanical system in which these concepts are investigated is shown in Fig. 1 (see Materials and Methods), and it consists of two strongly coupled mechanical beams that sustain symmetric (*S*) and asymmetric (*A*) vibration modes at ω_*S*_/2π ≈ 298.4 kHz and ω_*A*_/2π ≈ 310.3 kHz with bandwidths of Δω_*S*_/2π ≈ 130 Hz and Δω_*A*_/2π ≈ 138 Hz, respectively (*15*). The mechanical elements are integrated with piezoelectric transducers, which enable the underlying harmonic potential of both modes to be parametrically modulated, leading to a system Hamiltonian given by

$$H=\sum_{n=S}^{A}(p_{n}^{2}/2+\omega_{n}^{2}q_{n}^{2}/2(1-2\Gamma_{n}\;\text{cos}(2\omega_{n}t)+\beta_{n}q_{n}^{2}/2))+\Lambda q_{S}q_{A}\;\text{cos}(\omega_{𝒫}t+\phi )$$ (1)

where the summation expresses the kinetic and potential energies from both modes in terms of their position *q*_*n*_ and canonically conjugate momentum *p*_*n*_ (*15*–*17*). The potential energy term contains three contributions, with the second contribution arising from the periodic modulation of the mechanical spring constant with amplitude Γ_*n*_ at twice the natural mode frequency, which yields degenerate parametric amplification and parametric resonance (*18*, *19*), and the third contribution arising from the Duffing nonlinearity β_*n*_, a well-known anharmonicity that emerges at large displacements (*20*). Γ_*n*_ can be experimentally activated by modulating the spring constant of either mode with the application of voltage *V*_*n*_(2ω_*n*_) to induce stress from the piezoelectric transducers, and at sufficiently large amplitude, it results in a parametric resonance as detailed in the Supplementary Materials (*16*, *19*). Projecting the parametric resonances into phase space under pulsed driving, via the demodulation circuit in Fig. 1 (which records the in-phase *Q*_*n*_ and quadrature *P*_*n*_ components of position), reveals that they can vibrate with only two phases separated by π radians, as shown in Fig. 2 (A and B) (*16*, *21*). These phase bistable vibrations provide the ideal means to encode a classical spin in the mechanical domain where the positive in-phase component is defined as spin up, that is, σ_*n*_ = 1, and vice versa as depicted by the arrows in Fig. 2 (A and B) (*8*, *12*).

The last term in Eq. 1 describes nondegenerate parametric down-conversion from the pump 𝒫 with amplitude Λ at the sum frequency of both modes $\left(\omega_{𝒫}=\omega_{S}+\omega_{A}\right)$, which results in the symmetric and asymmetric modes becoming correlated, yielding a two-mode squeezed state (*15*). Λ can be experimentally activated by piezoelectrically pumping the spring constant of both modes with voltage $V_{𝒫}(\omega_{𝒫})$, which simultaneously amplifies their thermomechanical fluctuations, as shown in Fig. 2 (C and D) (*22*). The concurrent generation of phonons in this process leads to the vibrations of both modes becoming correlated, which can be identified by reconstructing their cross-quadratures in phase space, namely, the in-phase component of the asymmetric mode versus the quadrature component of the symmetric mode and vice versa, as shown in Fig. 2 (E and F). The resultant squashed distributions in phase space imply that the motion of both modes is perfectly intertwined and is statistically confirmed by their unity correlation coefficient as detailed in the Supplementary Materials (*15*).

**Fig. 3:**
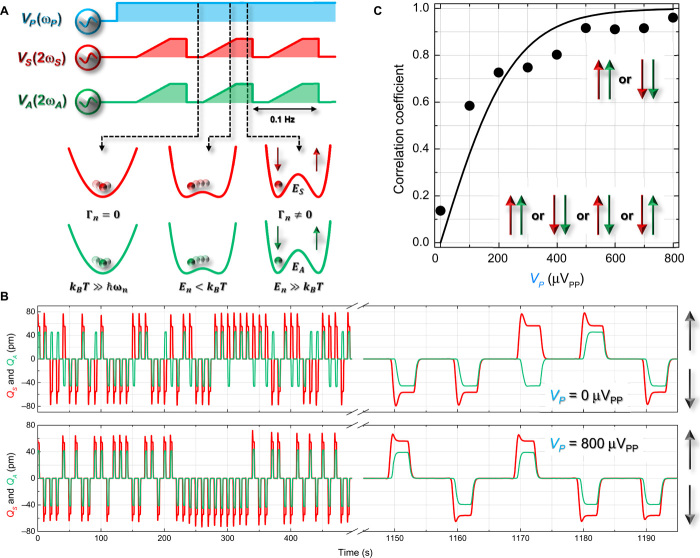
Mechanical spin coupling. (**A**) The pulse sequence used to demonstrate the fundamentals of the Ising Hamiltonian in the electromechanical system and the corresponding qualitative evolution of the underlying potentials from both modes. The correlations generated by $V_{𝒫}(\omega_{𝒫})$ can be visualized as the synchronous motion of the balls in the harmonic potentials of the symmetric (red) and asymmetric (green) modes. As *V*_*S*_(2ω_*S*_) and *V*_*A*_(2ω_*A*_) are slowly ramped, the harmonic potentials start to evolve into double-well potentials where the thermal energy in the system (in addition to dissipation) can drive transitions between the two oscillation phases. Finally, when both modes are parametrically resonating, their vibrations are correlated (that is, they occupy the same potential minima) and frozen where the thermal fluctuations are too small to destroy the phase of the vibrations. (**B**) The temporal response of both modes to the above pulse sequence with *V*_*S*_(2ω_*S*_) = 1.6 V_PP_ and *V*_*A*_(2ω_*A*_) = 2.1 V_PP_ when $V_{𝒫}(\omega_{𝒫})=0V_{\text{PP}}$ and 800 μV_PP_ with the mechanical spin orientations visualized by the arrows. (**C**) The correlation coefficient extracted from the temporal response of both modes as a function of $V_{𝒫}(\omega_{𝒫})$ (points) reveals a ferromagnetic state at large pump amplitudes, but as it is reduced to zero, the spins become disordered with a profile that is consistent with the Ising interaction between a pair of spins (line) as detailed in Materials and Methods.

These observations suggest that correlations generated between the two harmonic modes from nondegenerate parametric down-conversion, as schematically depicted in Fig. 3A with Γ_*n*_ = 0, and the double-well potential underpinning the phase bistable vibrations of a parametric resonance (*17*, *23*) provide all the ingredients necessary to realizing a phonon-based simulator for the Ising Hamiltonian. The key to implementing this vision is the ability to generate correlations between the double-well potentials underlying the parametric resonances from both modes through parametric down-conversion as visualized in Fig. 3A with Γ_*n*_ ≠ 0.

First, to theoretically confirm the viability of this approach, the above Hamiltonian is transformed in the rotating-frame approximation with the introduction of a new canonical position coordinate *Q*_*n*_ and a conjugate momentum *P*_*n*_, as detailed in Materials and Methods, which yields

$$H=\sum_{n=S}^{A}-E_{n}+J_{SA}\sigma_{S}\sigma_{A}$$ (2)

where the first term describes the quasi-energy $E_{n}=\omega_{n}\Gamma_{n}^{2}/6\beta_{n}$ separating the two stable oscillation phases of the parametric resonances at *P*_*n*_ = 0 and $Q_{n}=\sigma_{n}\sqrt{4\Gamma_{n}\omega_{n}/3\beta_{n}}$ with their two phases encoding σ_*n*_ = ±1 (see the Supplementary Materials) and the second term quantifies the coupling $J_{SA}=\Lambda \;\text{cos}(\phi )\sqrt{\Gamma_{S}\Gamma_{A}/{9\beta }_{S}\beta_{A}}$ between the modes. This electromechanical system can therefore be formally mapped onto the Ising Hamiltonian composed of two particles with spins corresponding to the phase bistable parametric resonances of both modes, which can be coupled via nondegenerate parametric down-conversion. This analysis also reveals that the polarity of the coupling can be tuned by the pump phase ϕ (*24*).

### Experimental implementation and analysis

To verify this concept, the protocol depicted in Fig. 3A is developed where a two-mode thermally squeezed state is initially created with $V_{𝒫}(\omega_{𝒫})\neq 0$ as visualized by the correlated fluctuations of the balls (signifying mechanical motion) in the harmonic potentials of the symmetric and asymmetric modes. Next, both modes are simultaneously and slowly (that is, << Δω_*S*_/2π and Δω_*A*_/2π) activated via *V*_*n*_(2ω_*n*_), which results in their harmonic potentials evolving to the double-well potentials of their parametric resonance, as shown in Fig. 3A with Γ_*n*_ ≠ 0, where the balls have now stabilized in one of the two potential minima corresponding to either a spin up or a spin down. In the slow transition from the harmonic to the double-well potentials, an intermediate regime exists where thermal fluctuations (in addition to dissipation) can drive transitions between the two oscillation phases, namely, *E*_*n*_ < *k*_B_*T*, where *k*_B_ is the Boltzmann constant and *T* is the temperature, which can stimulate the search for the global potential minima in an electromechanical Ising simulator. The spin information is then deleted by deactivating both parametric resonances, and the protocol is repeated for 2000 s. The spin information encoded in the two modes is identified by the polarity of the in-phase component of their parametric resonance, and it yields a train of switching outputs from both modes, with a period defined by this sequence, namely, 0.1 Hz, as shown in Fig. 3A. Implementation of this protocol over this duration provides a statistical ensemble from which the nature of the correlation between the two mechanical spins can be reliably and quantitatively evaluated.

Experimentally, in the case when $V_{𝒫}(\omega_{𝒫})=0V_{\text{PP}}$ (peak-to-peak voltage), the outputs from the symmetric and asymmetric modes reveal spin polarities, which are independent of each other, implying the absence of coupling between them or, in other words, *J*_*SA*_ = 0 as shown in Fig. 3B. The corresponding correlation coefficient extracted from this measurement is almost 0, as shown in Fig. 3C, which quantitatively confirms this observation. On the other hand, implementing this sequence with $V_{𝒫}(\omega_{𝒫})=800\mu V_{\text{PP}}$ yields the output shown in the lower panel of Fig. 3B, which reveals that the mechanical spins always exhibit parallel alignment, namely, ferromagnetic coupling with *J*_*SA*_ >> *k*_B_*T*. The corresponding correlation coefficient confirms this observation, yielding a value of almost 1, as shown in Fig. 3C. Next, implementing this protocol as a function of pump amplitude and extracting the resultant correlation coefficients yield the response shown in Fig. 3C. This indicates that the ferromagnetic state can be controllably created by the pump, with the corresponding correlation coefficient smoothly transitioning from 0 to 1 with a profile that is consistent with correlations between two hypothetical spins interacting via the Ising Hamiltonian (as detailed in Materials and Methods and shown by the solid line in Fig. 3C). Consequently, $V_{𝒫}(\omega_{𝒫})$, namely, Λ, can convincingly play the role of *J*_*SA*_ in the electromechanical Ising simulator.

**Fig. 4:**
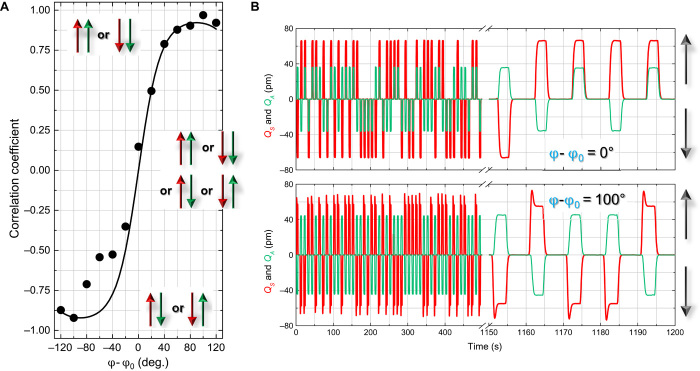
The Ising Hamiltonian replicated with electromechanical phonons. (**A**) The correlation coefficient extracted from the electromechanical system’s temporal response to the pulse sequence in Fig. 3A as a function of pump phase (points) with $V_{𝒫}(\omega_{𝒫})=800\mu V_{\text{PP}}$. (**B**) As the phase is adjusted, the correlated mechanical spins corresponding to a ferromagnetic state smoothly transition first to a disordered state without any coupling corresponding to random spin alignment (upper panel) and then to an anticorrelated state with the spins exhibiting antiferromagnetic ordering (lower panel), with this variation being consistent with the spin Ising interaction (line) as detailed in Materials and Methods.

To control the sign of *J*_*SA*_ in the electromechanical Ising simulator, ϕ is adjusted as suggested by Eq. 2 and experimentally depicted in Fig. 1 (see the Supplementary Materials). To this end, the protocol outlined in Fig. 3A is reimplemented but now as a function of ϕ with $V_{𝒫}(\omega_{𝒫})=800\mu V_{\text{PP}}$, which ensures that the mechanical spins are perfectly coupled, as shown in Fig. 3C. The results of this measurement in terms of the extracted correlation coefficient are shown in Fig. 4A, which reveal that it can be continuously tuned from 1 to −1 crossing 0, where the latter two time series are shown explicitly in Fig. 4B. In other words, starting from a ferromagnetic state, the mechanical spins transition to an antiferromagnetic state via an uncoupled state; that is, the *J*_*SA*_ → −*J*_*SA*_ operation can be executed on demand via ϕ.

## DISCUSSION

Electro-optomechanical systems (*10*, *25*) have emerged as a versatile platform where ultra-precise sensors can be developed (*26*, *27*), dynamically engineered nonlinearities can be harnessed (*28*, *29*), and quantum mechanics within a macroscopic context can even be studied (*30*–*33*). The notion of solving mathematical problems with phonons localized in spectrally pure resonances, which is advanced here, offers a new chapter to the mechanical resonator narrative.

**Fig. 5:**
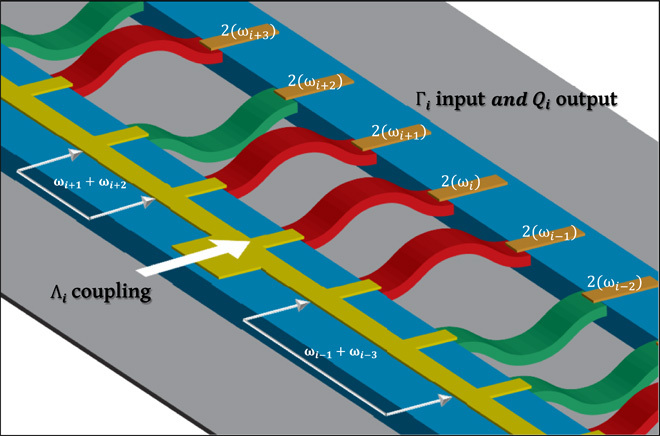
The electromechanical simulator. A conceptual image of an electromechanical Ising machine based on phonons confined in an array of mechanical elements each parametrically resonating to encode classical spin information (red corresponds to spin up and green corresponds to spin down) where two-mode squeezing is used to create coupling between elements. The parametric resonances are piezoelectrically activated and read out via the individual gate electrodes (orange) on each mechanical element where the *i*th element has a natural frequency ω_i_. The global gate electrode (yellow) located on the left clamping point of all the mechanical elements can enable coupling between any pair of mechanical spins with the application of a sum frequency pump. Using FDM, the pump can execute multiple degrees of coupling between large numbers of spins, potentially enabling the Ising Hamiltonian to be explored in a nontrivial configuration.

The results detailed in Figs. 3 and 4 confirm that the requisite features of the Ising Hamiltonian can be reproduced by phonons sustained by an electromechanical system. However, this implementation with two mechanical modes is trivial, and therefore, it is instructive to examine the prospects of a more useful Ising machine based on these ideas, as visualized in Fig. 5. Here, an array of mechanical elements with different frequencies are weakly mechanically coupled to their neighbors (*34*). The elements encode spin information via the bistable phase of their piezoelectrically activated parametric resonance in their fundamental mode, via the right clamping point, from where this information can be programmed and read out (*16*). The key difference here is that spin information stored in a given mode is concentrated within each mechanical element in contrast to the above demonstration. On the left clamping point, a coupling gate electrode is defined, which interconnects the mechanical spins via piezoelectrically activated parametric down-conversion at the sum frequency of two elements, for instance, $\omega_{𝒫}=\omega_{i-1}+\omega_{i-3}$. Naturally, the reduced mechanical coupling between the elements will require a more intense pump to compensate, which is readily available to this architecture (*15*). Uniquely, in this scheme, each mechanical spin can then be easily coupled to all its neighbors by using a frequency division–multiplexed (FDM) pump composed of multiple sum frequencies, where the availability of arbitrary coupling between the mechanical elements via the FDM pump is depicted in Fig. 5. The compact and highly flexible form of this coupling is in stark contrast to the optical Ising machine, which requires delay lines that increase both in number for more spins and in length for higher-order couplings (*9*). The FDM piezoelectric pump, in principle, can enable a large number of mechanical spins to sustain multiple degrees of coupling, thus permitting the electromechanical Ising simulator to be programmed to explore problems that challenge conventional computers. However, in practice, the ultimate number of spins, with maximal coupling (that is, *N*^2^), will be limited by the global coupling gate’s ability to sustain the sum of the *N*^2^ coupling voltages before its Schottky barrier breaks down and neutralizes the piezoelectric transduction.

Although this platform could probe NP-hard Ising problems (*8*, *9*), it does not offer speed-up because it uses classical annealing where the thermomechanical fluctuations of the mechanical elements drive the search in the underlying potential energy landscape for the ground state. However, if all the mechanical elements can be operated in their ground state, then quantum effects could be potentially harnessed to explore the possibility of increasing the speed of search (*30*–*32*). Alternatively, the universal nature of this concept allows for it to be exploited by any kind of resonator, even from superconductors that sustain both parametric resonances (*35*) and nondegenerate parametric down-conversion (*36*), thus enabling the quantum dynamics of this concept to be explored.

## MATERIALS AND METHODS

### Experimental

The electromechanical system was synthesized from a GaAs/AlGaAs heterostructure sustaining a 100-nm-thick, uniformly doped GaAs layer located 300 nm below the surface (shown in blue in Fig. 1) via conventional micromachining processes (*15*). The mechanical elements have a length, width, and thickness of 80 μm, 20 μm, and 800 nm, respectively, which are strongly intercoupled via two 40-μm-wide overhangs with the same thickness and a length of 16 μm. Piezoelectric transducers were incorporated into the clamping points of both mechanical elements, which are composed of the doped layer and gold electrodes sandwiching an insulating GaAs/AlGaAs superlattice.

The parametric resonances and nondegenerate parametric down-conversion were piezoelectrically activated with multiple AC signal generators (NF Wave Factory 1974) connected to the gold electrodes on the left element, as depicted in Fig. 1, which modulated the stress and hence the spring constant of the symmetric and asymmetric modes. The thermal motion and the parametric resonances of both modes were probed with a HeNe laser reflected from the right mechanical element with a 3-μW input and detected in a Doppler interferometer (Neoark MLD-221). The output from the interferometer was demodulated either in a spectrum analyzer (Agilent 89410A) or in two phase-sensitive detectors (Stanford Research Systems SR844) that were mixed with two local oscillators (NF Wave Factory 1974) locked onto the resonances of both modes.

### Theory

The Hamiltonian for a resonantly excited degenerate parametric oscillator with natural frequency ω_0_ can be expressed as (*37*)

$$H_{0}=\frac{p^{2}}{2}+\frac{1}{2}\omega_{0}^{2}q^{2}(1-2\Gamma \text{cos}(2\omega_{0}t)+\frac{\beta q^{2}}{2})$$ (3)

This Hamiltonian can be translated into the rotating frame following the standard procedure (*38*) with the introduction of canonically conjugate variables *P* and *Q* defined as

$$\begin{array}{c} q=(P\;\text{sin}(\omega_{0}t)+Q\text{cos}(\omega_{0}t))/\sqrt{\omega_{0}}\\ p=\sqrt{\omega_{0}}(P\;\text{cos}(\omega_{0}t)-Q\text{sin}(\omega_{0}t)) \end{array}$$ (4)

The effective Hamiltonian in these variables is then given by

$$H_{0}(P,Q)\sim \frac{\omega_{0}\Gamma }{4}(P^{2}-Q^{2})+\frac{3\beta }{32}{\left(P^{2}+Q^{2}\right)}^{2}$$ (5)

where all the off-resonant and rapidly oscillating terms have been omitted (*37*). This Hamiltonian has two minima, extracted via ∂H/∂P=∂H/∂Q=0, at

$$P=0,Q=\sigma \sqrt{4\Gamma \omega_{0}/3\beta },\sigma =\pm 1$$ (6)

separated by a saddle point at *P* = *Q* = 0 (see the Supplementary Materials). At steady state, the bistable oscillating states of the parametric resonator are energetically degenerate with $H_{0}=-\omega_{0}^{2}\Gamma^{2}/6\beta$, and they can mimic a classical two-level or a spin 1/2 system via *Q*, as experimentally confirmed in Fig. 2 (A and B).

The Hamiltonian for two degenerate parametric oscillators that are coupled via nondegenerate parametric down-conversion is given in Eq. 1, and it can also be translated into the rotating frame via Eq. 4, which yields

$$H(P,Q)\sim \sum_{n=S}^{A}(\frac{\omega_{n}\Gamma_{n}}{4}(P_{n}^{2}-Q_{n}^{2})+\frac{3\beta_{n}}{32}{\left(P_{n}^{2}+Q_{n}^{2}\right)}^{2})+\frac{\Lambda }{4\sqrt{\omega_{S}\omega_{A}}}((Q_{S}Q_{A}-P_{S}P_{A})\text{cos}(\phi )-(P_{S}Q_{A}+Q_{S}P_{A})\;\text{sin}(\phi ))$$ (7)

If the nondegenerate parametric coupling strength Λ is weak (that is, in the amplification regime as detailed in fig. S1C), then the steady state in Eq. 6 can be used to approximate the lowest-order solution yielding Eq. 2.

### Correlations between spins

The theoretical correlations between two spins, whose interaction is mediated by the Ising Hamiltonian, were evaluated to verify the experimental variation of the correlation coefficient as a function of pump amplitude and phase to confirm the viability of the electromechanical simulator. For a pair of spins, the probability of finding a particular spin configuration is given by

$$P(\sigma_{S}\sigma_{A})=\frac{1}{Z}\;\text{exp}(-\alpha J_{SA}\sigma_{S}\sigma_{A})$$ (8)

where the partition function is defined as

$$Z=\sum_{\sigma_{S}\sigma_{A}}\;\text{exp}(-\alpha J_{SA}\sigma_{S}\sigma_{A})=4\;\text{cosh}(\alpha J_{SA})$$ (9)

with α = 1/*k*_B_*T*. The correlation between nearest-neighbor spins can then be expressed as

$$\langle \sigma_{S}\sigma_{A}\rangle =\sum_{\sigma_{S}\sigma_{A}}\sigma_{S}\sigma_{A}P(\sigma_{S}\sigma_{A})=\;\text{tanh}(\alpha J_{SA})$$ (10)

where this metric is analogous to the correlation coefficient. This equation can reproduce the experimental response using a fitting parameter defined as $\alpha J_{SA}/V_{𝒫}(\omega_{𝒫})=4000V^{-1}$, which yields the line in Fig. 3C. This analysis reveals that as the pump intensity is increased, a ferromagnetic state can be created, the activation of which is consistent with the Ising Hamiltonian where the correlation generated via parametric down-conversion competes with the random thermal fluctuations and is balanced at $V_{𝒫}(\omega_{𝒫})=250\mu V_{\text{PP}}$, where *J*_*SA*_ = *k*_B_*T*. A similar analysis can also be performed as a function of pump phase with *J*_*SA*_cos(ϕ), yielding 〈σ_*S*_σ_*A*_〉 = tanh(α*J*_*SA*_cos(ϕ)), which reproduces the experimental result shown by the line in Fig. 4A.

## Supplementary Material

http://advances.sciencemag.org/cgi/content/full/2/6/e1600236/DC1

## SUPPLEMENTARY MATERIALS

Supplementary material for this article is available at http://advances.sciencemag.org/cgi/content/full/2/6/e1600236/DC1 I. Degenerate and nondegenerate parametric amplification II. The double-well potential III. Pump phase fig. S1. Experimentally measured degenerate and nondegenerate parametric amplification of both modes in the electromechanical system. fig. S2. The double-well potential underpinning a parametric resonance. fig. S3. The pump phase dependence of the two-mode squeezing.
