# Supplementary material for: An electromechanical Ising Hamiltonian
Source: Sci Adv. 2016 Jun 24;2(6):e1600236. doi: 10.1126/sciadv.1600236 (PMC5566114; doi:10.1126/sciadv.1600236)
Supplement: http://advances.sciencemag.org/cgi/content/full/2/6/e1600236/DC1 [file supp_2_6_e1600236__index.html]

Science Advances | Science Advances

## Supplementary Materials

**This PDF file includes:**

- I. Degenerate and nondegenerate parametric amplification
- II. The double-well potential
- III. Pump phase
- fig. S1. Experimentally measured degenerate and nondegenerate parametric amplification of both modes in the electromechanical system.
- fig. S2. The double-well potential underpinning a parametric resonance.
- fig. S3. The pump phase dependence of the two-mode squeezing.

Download PDF

**Files in this Data Supplement:**

- Adobe PDF - 1600236\_SM.pdf
