# Supplementary material for: An electromechanical Ising Hamiltonian
Source: Sci Adv. 2016 Jun 24;2(6):e1600236. doi: 10.1126/sciadv.1600236 (PMC5566114; doi:10.1126/sciadv.1600236)
Supplement: http://advances.sciencemag.org/cgi/content/full/2/6/e1600236/DC1 [file 1600236_SM.pdf]

Science Advances

AAAS

advances.sciencemag.org/cgi/content/full/2/6/e1600236/DC1

Supplementary Materials for

An electromechanical Ising Hamiltonian

Imran Mahboob, Hajime Okamoto, Hiroshi Yamaguchi

Published 24 June 2016, *Sci. Adv.* **2**, e1600236 (2016)
DOI: 10.1126/sciadv.1600236

This PDF file includes:

- I. Degenerate and nondegenerate parametric amplification
- II. The double-well potential
- III. Pump phase
- fig. S1. Experimentally measured degenerate and nondegenerate parametric amplification of both modes in the electromechanical system.
- fig. S2. The double-well potential underpinning a parametric resonance.
- fig. S3. The pump phase dependence of the two-mode squeezing.

I. Degenerate and nondegenerate parametric amplification

Degenerate parametric amplification can be activated in the electromechanical system by piezoelectrically modulating the spring constant of either mode at twice their natural frequency via $V_n(2\omega_n)$ in Fig. 1 namely Γ_n in equation 1. This process amplifies the thermally driven random displacement fluctuations of either mode as shown in fig. S1, A and B and the corresponding gain is accompanied by a reduction in their damping rates. Once the phonon generation rate from this process exceeds the rate of loss from either mode, it results in parametric resonance which yields an oscillation with a bistable phase (*16*).

Nondegenerate parametric amplification can also be activated in the electromechanical system by piezoelectrically modulating the spring constant at the sum frequency of both modes via $V_{\mathcal{P}}(\omega_{\mathcal{P}})$ in Fig. 1 namely Λ in equation 1. In contrast to the degenerate parametric amplification, this process *simultaneously* amplifies the random displacement fluctuations of *both* modes as shown in fig. S1C and is also accompanied by a reduction in their damping rates (*15*).

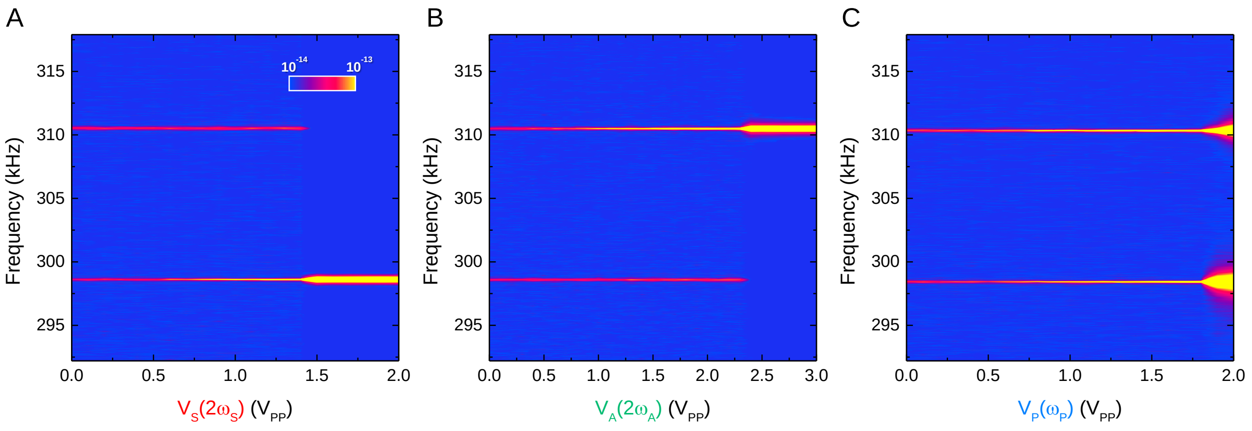

fig. S1. Experimentally measured degenerate and nondegenerate parametric amplification of both modes in the electromechanical system. (A-C) The thermal motion of the symmetric and asymmetric modes as they are degenerately and nondegenerately amplified when the electromechanical system's spring constant is piezoelectrically modulated at either twice the frequency of the symmetric or asymmetric mode or at their sum frequency respectively. The logarithmic spectral displacement density scale (mHz$^{-0.5}$) is identical in all figures.

II. The double-well potential

The Hamiltonian in equation 5, describing a single degenerate parametric resonator excited on resonance, can be plotted via the canonically conjugate coordinates P and Q with $\omega_0 = 1$, $\beta = 1$ and $\Gamma = 1$ as shown in fig. S2. This plot reveals a symmetric double well potential underpinning the parametric resonance where the two minima correspond to the two stable oscillation phases, separated by a quasi-energy E_n, which are used to encode mechanical spins namely σ_n (*37*).

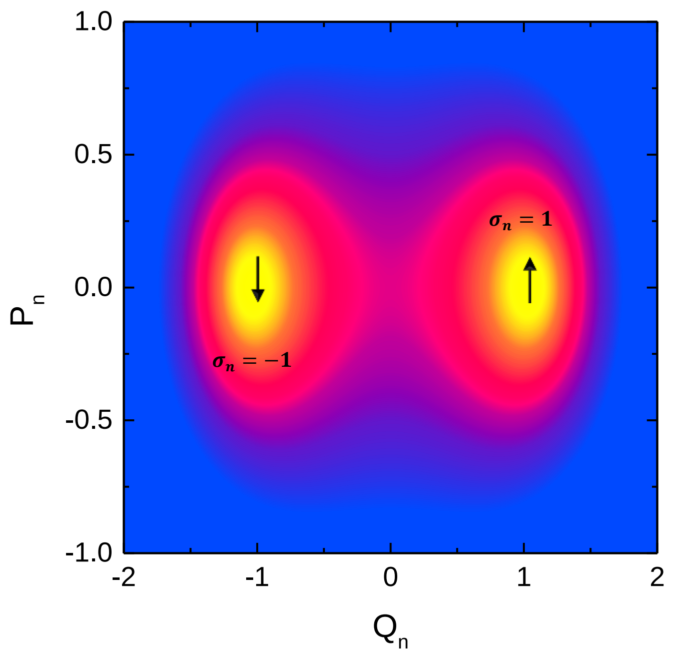

fig. S2. The double-well potential underpinning a parametric resonance. The double-well potential of a parametric resonance can be used to mimic spin 1/2 particles with an up spin i.e. $\sigma_n = 1$ corresponding to a positive Q_n coordinate and vice versa.

III. Pump phase

The correlations generated in the two-mode squeezed states from the nondegenerate parametric down-conversion can be tuned via the pump phase φ as observed in other physical implementations (*24*). Here this effect is employed to control the sign of the coupling constant J_{SA} between the two mechanical spins as detailed in equation 2 and Fig. 4A. To understand the nature of this phenomenon, the polarity of the correlations as a function of φ are measured from the thermal Langevin force driven displacement fluctuations of both modes whilst they are nondegenerately amplified (*15*). At each pump phase, the correlation coefficients from the cross-quadratures Q_S: P_A and Q_A: P_S are extracted as detailed in fig. S3A. This reveals that a correlated state with unity correlation coefficient (upper panels in fig. S3B) can be tuned to an uncoupled or random configuration with zero correlation coefficient (middle panels in fig. S3B) and to an anti-correlated state with a correlation coefficient of -1 (lower panels in fig. S3B). These observations indicate that by conditioning the two-mode thermal squeezed states via φ enables the creation of a ferromagnetic or an anti-ferromagnetic state on-demand in the electromechanical Ising simulator.

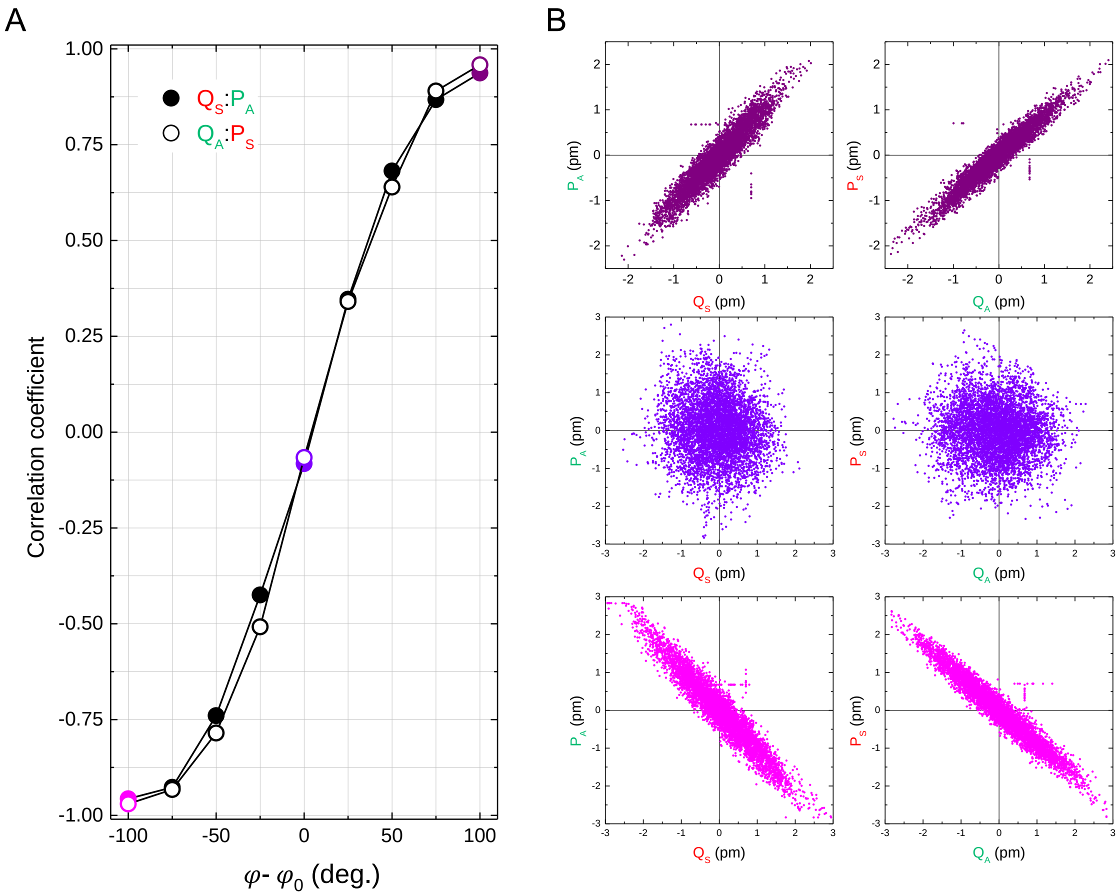

fig. S3. The pump phase dependence of the two-mode squeezing. (A) The correlation coefficient extracted from the cross-quadratures $Q_S\!:P_A$ and $Q_A\!:P_S$ with $V_{\mathcal{P}}(\omega_{\mathcal{P}}) = 1.3$ V_{PP} as a function of φ. **(B)** The phase portraits reconstructed from the cross-quadratures of the symmetric and asymmetric modes with the pump phase detailed via the color coding in **A**. As the pump phase is adjusted, the two-mode squeezed states can be continuously tuned from a positively correlated to an anti-correlated state via a random state.
